# The Effect of Wood Ash as a Partial Cement Replacement Material for Making Wood-Cement Panels

**DOI:** 10.3390/ma12172766

**Published:** 2019-08-28

**Authors:** Viet-Anh Vu, Alain Cloutier, Benoit Bissonnette, Pierre Blanchet, Josée Duchesne

**Affiliations:** 1 Department of Wood and Forest Sciences, Laval University, Quebec, QC G1V 0A6, Canada; 2 Department of Civil Engineering, Laval University, Quebec, QC G1V 0A6, Canada; 3 Department of Geology and Geological Engineering, Laval University, Quebec, QC G1V 0A6, Canada

**Keywords:** biomass, wood ash, fibrocement, strength, mortar

## Abstract

The aim of this study was to consider the use of biomass wood ash as a partial replacement for cement material in wood-cement particleboards. Wood-cement-ash particleboards (WCAP) were made with 10%, 20%, 30%, 40%, and 50% of wood ash as a partial replacement for cement with wood particles and tested for bending strength, stiffness, water absorption, and thermal properties. Test results indicate that water demand increases as the ash content increases, and the mechanical properties decrease slightly with an increase of the ash content until 30% of replacement. On the other hand, the heat capacity increases with the wood ash content. The WCAP can contribute to reducing the heat loss rate of building walls given their relatively low thermal conductivity compared to gypsum boards. The replacement of cement to the extent of approximately 30% by weight was found to give the optimum results.

## 1. Introduction

Fiber cement panels have been on the market for a long time. Originally, asbestos was used as the reinforcing material, but due to the health hazards involved, it was replaced by cellulose in the 1980s. Nowadays, these panels are used as exterior siding, roof shingles, and tiles for exterior applications. Wood-cement particleboard has several benefits, since it is resistant to termites, does not rot, is impact resistant, and has fireproof properties. However, studies carried out on the compatibility of wood with cement [1,2,3] show that not all species are equally suited for the manufacturing of wood-cement particleboard. Softwood species actually show the greatest potential for this type of application. The results of Tittelein et al. [4] show that it is possible to make low-density (specific gravity of about 0.7) wood-cement particleboards with better bending properties than gypsum boards and a screw-withdrawal resistance that is 1.7 times higher. Moreover, these panels can be cut with a knife in a similar manner as gypsum boards. Therefore, the panel installation process is essentially the same. Thanks to its high porosity, the thermal conductivity of wood-cement particleboards is about three times lower than that of gypsum boards.

Environmental concerns and economic pressure are amongst the driving forces of today’s industrial development. Therefore, several research projects are being conducted worldwide on the use of waste materials to reduce threats to the environment and to streamline present waste disposal and recycling methods by making them more affordable [5].

Manufacturing of ordinary Portland cement (OPC) ranks third in the world among the producers of anthropogenic CO_2_, after transport and power generation. The emission of CO_2_ by the cement industry represents 5%–7% of the total worldwide CO_2_ emissions from fuel combustion and industrial activities [6]. The use of additives and substitutes to OPC has been so far one of the most successful solutions to decrease CO_2_ emission generated by cement production.

Wood ash (WA) is produced by the combustion of wood in domestic wood stoves or in industrial power generation plants. At the end of the 80s, an estimated quantity of 45,000 tons of wood ash was produced annually in the Province of Québec, Canada by the pulp and paper industry [7]. In 2006, more than 300,000 tons of wood ash were produced per year, two-thirds coming from pulp and paper plants and the remaining from cogeneration plants, sawmills, and other wood-related industries. WA chemical characteristics differ with species of wood, but it mainly contains lime and silica [8]. Ash production is likely to expand further with the increasing interest for bioenergy.

In 2007, 150,000 tons of residual ash were used as fertilizers in Quebec [9]. Most of the residual ash (54%) was used in agriculture. The rest was used for the revegetation of degraded sites, soil mix manufacturing, composting, and other uses. Half of the wood ash resource produced annually is still landfilled [9]. When favorable conditions are met, the wood ash may have some pozzolanic potential that can be taken advantage of in Portland cement-based systems.

Several studies have investigated the suitability of wood ash as a supplementary cementing material in the production of ordinary and self-compacting concretes. Subramaniam [10] reported an optimum dosage of wood ash of 15% in the replacement of cement (by weight) for the production of concrete having a sufficiently high compressive strength for the casting of blocks. Abdulladi [11] found an optimum replacement rate of 20% and showed that the water requirement increases as the wood ash content increases. Chowdhury et al. [12] characterized the mechanical strength (compression, tensile, and flexural) of concrete incorporating wood ash. The presence of essential pozzolanic compound (as required by the ASTM C618-15 standard), the content in small size particles, and the large surface area of the particles qualify the wood ash investigated in their study as a pozzolanic material.

The aim of the present study was to evaluate the physical, thermal, and mechanical properties of wood-cement particleboards prepared using wood ash as a supplementary cementing material.

## 2. Materials and Methods

### 2.1. Materials

The main binder used was an ordinary CSA (Canadian Standards Association) type 10 (GU, General Use) Portland cement.

The wood ash selected for investigation was supplied from the thermal energy production plant of “La Cité Verte”, a residential development in Quebec, QC, Canada.

The wood-cement mixtures were prepared with air-dried wood chips obtained from white spruce (*Picea glauca* (Moench), Voss, Norway) trees harvested at the Petawawa Research Forest in Mattawa (ON), Canada. The wood chips were refined in a Pallmann PSKM8-400 ring refiner (Ludwig Pallmann K.G, Zweibrücken, Germany). The particles supplied were screened, and those ranging between 1 and 3 mm in size were retained.

### 2.2. Wood-Cement Mixtures

The wood-cement particleboard mixtures were all prepared with a wood-to-binder ratio of 0.35 by weight, where the binder phase is the sum of cement and wood ash. A total of six mixtures were investigated, the variables being essentially the fraction of cement replaced by wood ash. Assessing mixtures with different percentages of wood ash was intended to determine the maximum amount of wood ash that could be used without significantly affecting the properties of the material in comparison with those of the reference wood-cement mixture. The corresponding mixtures are referred to as P0, P1, P2, P3, P4, and P5, respectively. The control mixture (P0) was prepared with cement and wood particles only, while mixtures P1, P2, P3, P4, and P5 were prepared by incorporating wood ash as a partial replacement of cement at a rate of 10%, 20%, 30%, 40%, and 50%, respectively.

The mixing sequence was observed to have a critical influence upon the material rheology, with slight changes altering the fresh mixture behavior significantly. The mixing sequence retained after the preliminary tests is presented in Table 1.

Directly after mixing, the workability of each mixture was determined using the slump test in accordance with the ASTM C143/C143M-15a standard [13].

### 2.3. Preparation of Test Specimens

After mixing in a mortar mixer (HOBART A-120, Hobart Canada Inc, Don Mills, ON, Canada), each wood-cement-ash-water mixture was cast into a 450 × 330 × 15 mm wooden mold. After pouring the mixture, the mold was closed with a lid held in place by C-clamps. This set-up allowed to pour material up to a thickness of 15 mm. The wet mixture was poured into the mold, the surface was levelled off with a wood screed and the lid was finally secured in place. From the pressure of the lid, the panel thickness was reduced to 14 mm. The hardened panels were stripped from the mold at the age of 3 days and then stored in a conditioning chamber at 23 °C and 60% relative humidity. The various test specimens were cut from the panels (3 panels per mixture) on the day of testing.

### 2.4. Test Methods

The panels were cured and tested to determine their mechanical performance after 3, 7, and 28 days of curing following the ASTM D 1037-12 standard [14]. The bending modulus of rupture (MOR) and the modulus of elasticity (MOE) were determined at the same ages by MTS QTest-5 Universal Test Frames (MTS systems corporation, Eden Prairie, MN, USA) featuring The Elite Modular Control System. Screw-withdrawal resistance, water absorption, and thickness swelling were also tested following the ASTM D 1037-12 standard [14]. The thermal properties of the wood-cement particleboards were measured by FOX 314 Heat Flow Meter (TA instruments-LaserComp Inc, Wakefield, MA, USA) following the ASTM C518 [15] standard. The board was placed between two plates at a regulated temperature and a flux meter was glued on each side of it so that temperature and heat flux could be measured at the board surface, which can be submitted to temperature variations. Heat capacity and thermal conductivity can be calculated from these four parameters (two temperatures and two heat fluxes). The solubility of the WA was evaluated by the mass loss measured on 15 g of WA placed in 100 mL of distilled water and stirred for one hour at 23 °C. The residue is then filtered under vacuum and rinsed with distilled water. The residue of WA is placed in an oven overnight then the loss of mass is measured. The soluble proportion corresponds to the average mass loss of tree samples. Finally, solid samples were observed under a JEOL JSM-840A Scanning Electron Microscope (JEOL USA Inc, Peabody, MA, USA) (SEM) equipped with an energy dispersive X-ray analysis system (EDS). For SEM observations, the specimens were mounted intact on double-sided adhesive tape and coated with a thin alloy of Au-Pd. Operating conditions were set at 15 kV.

## 3. Results

### 3.1. Material Characterization

#### 3.1.1. Wood Particles

**Figure 1:**
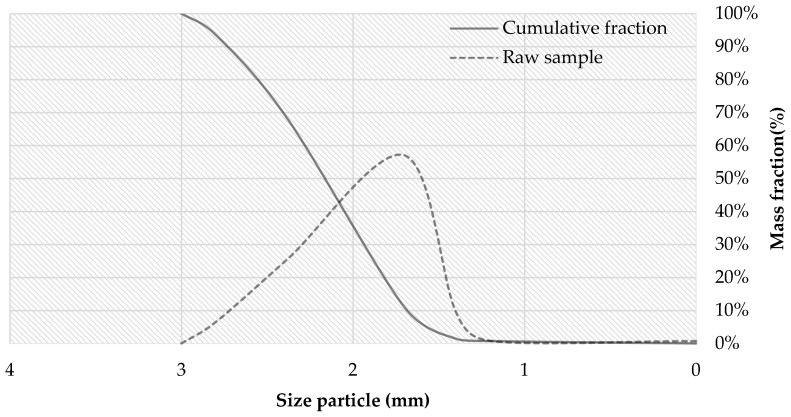
Particle size distribution of wood particles.

Wood particle size distribution was evaluated using five sieve sizes: 1.19, 1.4, 1.7, 2.38, 2.8, and 3 mm. According to the results shown in Figure 1, 100% of the particles were less than 3 mm in size, and particles with a diameter of 1.7 mm make for the highest mass fraction (57%).

#### 3.1.2. Wood Ash

##### Particle Size and Shape Analysis

**Figure 2:**
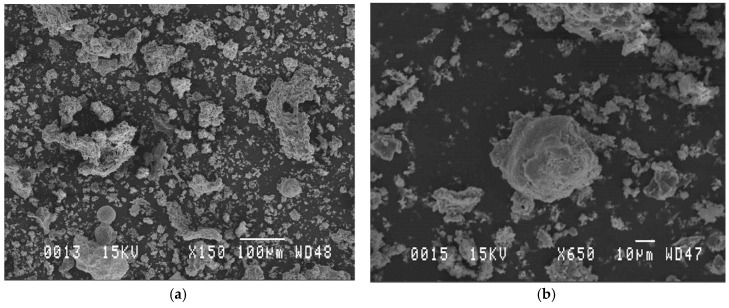
Low magnification (**a**) and high magnification (**b**) scanning electron microscopy of wood ash (WA).

**Figure 3:**
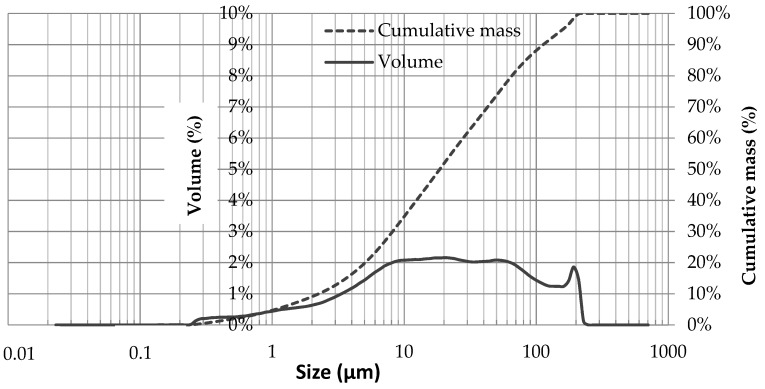
Particle size analysis of WA.

Shape analysis by scanning electron microscopy observations revealed that the ash particles were irregular in shape and spherical (Figure 2b). Wood ash is suitable for use as a filler/partial replacement of cement in high-performance concrete due to an enhanced “ball bearing” effect given from the spherical shape of WA. The “ball bearing” effect of wood ash creates a lubricating effect when concrete is in its plastic state. According to the results shown in Figure 3, the D10, D50, and D90 values of the WA were 2.5, 18.5, and 114.1 μm, respectively. Wood ash contains an amount of ultrafine particles of 18% (particle diameter ϕ < 5 μm).

##### Chemical Composition

The results of the chemical analysis carried out on the investigated wood ash are shown in Table 2. The combined content in iron oxide (Fe_2_O_3_ = 1.22%), aluminum oxide (Al_2_O_3_ = 2.25%), and silicium dioxide (SiO_2_ = 7.80%) is found to be 11.27%, which is considerably less than the minimum amount required to qualify a material as a pozzolan, established at 70% [16].

The recorded loss on ignition at 950 °C was 14.2%, which exceeds the 12% maximum requirement for pozzolans [16]. This means that the ash contains a significant amount of unburnt carbon, which reduces its pozzolanic activity. The alkali content (%Na_2_O + 0.658 × %K_2_O) was found to be 7.18%, a value higher than the maximum alkali content of 1.5% required for pozzolana. The specific gravity of wood ash was found to be 2.97, which is far less than the Portland cement density (3.15). WA contains more than 99% (by weight) of inorganic material and yields a pore solution with a high pH.

##### Solubility Test

Table 3 shows the percentage of the wood ash dissolved in water during the solubility test. The solubility of WA is estimated to be 7% including lime and alkali hydroxides that are readily soluble in water in laboratory conditions. This soluble component plays an important role in the hydration reaction.

### 3.2. Change in Density

The weight of all panels was recorded at the beginning and at the end of the curing period (3 days in the mold) to determine changes in the panel-specific gravity. It decreased by about 5% during that period, owing to the fact that the mold being used was not perfectly impervious. Some water was probably absorbed by the mold itself, as it was made of plywood.

The panel mass reached a plateau about 6 days after removal from the mold, meaning that most of the free water in the cement paste had evaporated in the conditioning chamber at 23 °C and 60% RH by then.

### 3.3. Workability

Table 4 shows the results obtained for the consistency test. The results reveal that the water demand increased with the wood ash content. The wood ash introduced into the cement increased the carbon content, thereby increasing the amount of water required to achieve satisfactory workability.

### 3.4. Bending Properties of the Raw Wood-Cement Particleboard

As described previously, the panels were tested in bending at 3, 7, and 28 days after manufacturing. Each test was performed on three specimens and the mean value is presented in Table 5.

**Figure 4:**
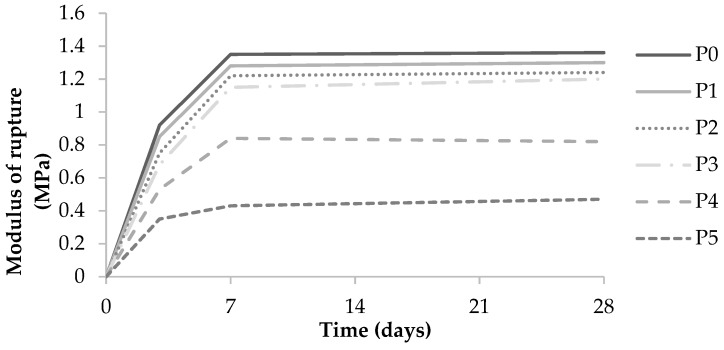
Evolution of the modulus of rupture in bending of WCAP as a function of the moist curing duration.

Table 5 and Figure 4 show the bending behavior of the WCAP at different curing times. It shows that the bending strength and stiffness values of the sample panels increase with the curing time. They changed little after 7 days of curing, as generally observed for Portland cement-based materials. The statistical analysis results showed that there is a significant difference among samples in terms of bending strength and stiffness at all stages of curing (3 days of curing: *p* < 0.001, 7 days of curing: *p* < 0.001, 28 days of curing: *p* < 0.05). The bending strength and stiffness of P4 and P5 were significantly lower than for the other panels at all curing stages. Optimum bending strength observed in these tests was obtained at 30% wood ash replacement (P3) after 28 days of moist curing.

### 3.5. Screw-Withdrawal Resistance

**Figure 5:**
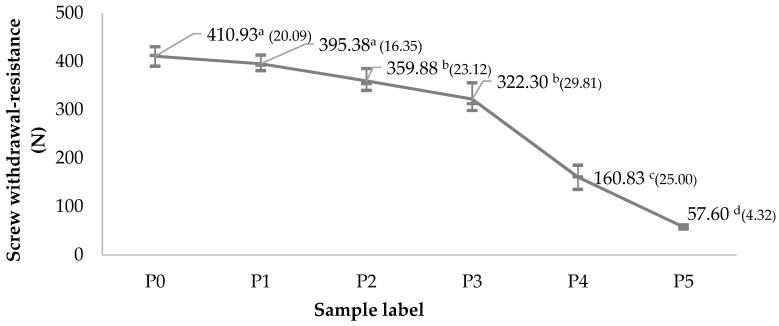
Effect of wood ash replacement rate on the screw-withdrawal resistance of the WCAP (mean values with the same superscript are not significantly different for *p* = 0.05; standard deviation is given in parentheses).

Figure 5 shows the screw-withdrawal resistance of WCAP as a function of the WA content. It shows that the screw-withdrawal resistance decreases as the WA replacement rate increases. The results of the statistical analysis show that the screw withdrawal-resistance is slightly affected up to a replacement rate of 30% in wood ash. However, beyond that value, it decreases rapidly.

### 3.6. Water Absorption

**Figure 6:**
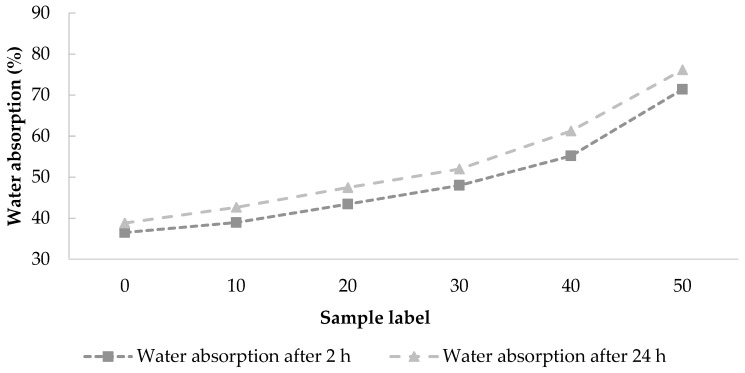
Water absorption and thickness swelling of WCAP recorded as a function of the WA content.

The water absorption test results are shown in Figure 6. The value of water absorption increases with the percentage of WA replacement and time of immersion in water. Table 6 shows that the thickness swelling of WCAP in water is small (<2%). According to the results, the water absorption of all boards incorporating wood ash is higher than that of the control sample after 28 days of curing.

### 3.7. Thermal Properties

Table 7 shows the WCAP heat capacity and thermal conductivity test results. It is interesting to note that the heat capacity increases with the wood ash content. It can contribute to reducing the heat loss rate of building walls given its relatively low thermal conductivity when used as interior partition. Wood ash level P3 yields a heat capacity 7% higher than that of the control panel. Conversely, the thermal conductivity does not change importantly between 0% and 30% wood ash replacement levels.

### 3.8. Microstructure of Mortars

**Figure 7:**
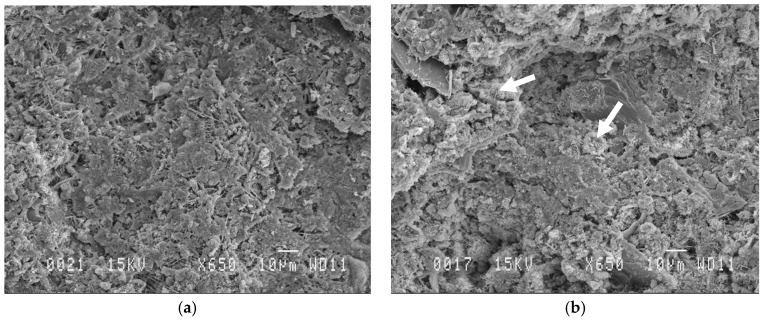
Scanning electron microscopy images of cement control (**a**) and cement + 30% WA (**b**).

According to the results shown in Figure 7, there are no clear differences in microstructure between the two samples. They both exhibit a low porosity and pore sizes smaller than 10 µm. The occurrence of spherical particles that have the shape of WA can be observed in Figure 7b as shown by the white arrows.

## 4. Discussion

Although the investigated wood ash does not qualify as a pozzolan, it can be used in replacement of cement up to significant amounts without affecting the physical and mechanical properties of the wood-cement particleboards significantly. In previous studies, maximum wood ash proportions in the order of 15%–20% were reported [10,11]. Compared to the control sample (P0), WCAP prepared with 30% of wood ash in replacement of cement (P3) showed moderate mechanical properties reductions of 10% for bending MOR and 21% for screw-withdrawal resistance. The pH value increases with the hydration of the cement. A high alkaline solution promotes the reactivity of the silica present in the WA, which enhances the pozzolanic activity at the initial stage. Increased pH levels favor the formation of hydrous silica. This compound reacts with Ca^2+^ ions and produces insoluble compounds, which are secondary cementitious products [10]. Moreover, WA can act as a filler in the mixtures.

The density of the samples is found to decrease as the WA replacement rate increases, due to the slightly lower density of the ash and, most importantly, the increased amount of water (Table 4 and Table 7). As a result of the larger volume of capillary pores, the mechanical and physical properties including density decline. Indeed, water absorption increased significantly from 30% of WA in replacement. It can be explained by the lower amount of cement particles with increasing wood ash contents. Therefore, the hydration reaction was reduced, and the water evaporated quickly in a porous medium with high porosity due to the presence of the wood fibers.

A fraction of the ash of about 7% dissolves in water and contributes to the hydration process. The large surface area associated to the ash particles could also be a factor, as it acts to some degree as nucleation sites for cement hydration. Indeed, based upon SEM examination, no significant difference in the microstructure of a mixture of neat cement and a mixture containing 30% of WA in replacement was found, both exhibiting a dense and uniform microstructure. 

The increase in the heat capacity of WCAP after replacement of cement by wood ash has shown that it has the potential to reduce the heat losses of building walls, given the improved insulation it provides. Indeed, WCAP has a low thermal conductivity, about three times lower than that of gypsum boards (0.32 W/m·K) [4]. This low thermal conductivity is mainly due to the higher WCAP porosity compared to that of gypsum because the thermal conductivity of empty voids is very low (about 0.025 W/m·K).

## 5. Conclusions

This project studied the physical, thermal, and mechanical properties of wood-cement particleboards incorporating wood ash. Wood ash was found to have an excellent potential for use as partial replacement to Portland cement. Based on the results generated in this study, the optimum replacement rate is about 30% by weight. At this replacement level, the engineering properties of WPCA were moderately reduced (bending MOR by 12%; bending MOE by 20%; screw-withdrawal resistance by 21%) compared to a neat wood-cement control sample. Beyond 30% in replacement, the mechanical and physical properties start to decrease at a significantly higher rate (bending MOR by 43%, bending MOE by 41%, and screw-withdrawal resistance by 60% at a 40% replacement rate). The use of wood ash improves the heat capacity of the WCAP by 11% compared to a neat wood-cement control sample.

The work reported herein is quite promising in view of producing eco-friendly wood cement panels with improved characteristics compared to those of standard gypsum boards. Future work should include the fire-resistance and acoustic properties measurement of this material. The formulation and the processing phases could also be further improved. Notably, the use of a paper surface layer should be studied to enhance the mechanical properties of the panel. 

## Figures and Tables

**Table 1:** Mixing sequence.

Step	Mixer Rotor Speed (rpm)	Cumulative Time (s)
1. Addition of cement and wood ash	140	0
2. Addition of water	140	60
3. Addition of wood particles	140	120
4. Change of speed	285	180
5. End of mixing	0	270

**Table 2:** Physical and chemical properties of wood ash.

Properties	Value	Chemical Composition (%)	
Conventional parameters		SiO_2_	7.80
Organic material (mg/kg)	<10	Al_2_O_3_	2.25
pH	13	Fe_2_O_3_	1.22
		MgO	7.47
Physical properties		CaO	46.70
Density (kg/m^3^)	2970	Na_2_O	0.86
Specific surface (m^2^/kg)	261	K_2_O	9.61
		TiO_2_	0.11
		MnO	4.51
		P_2_O_5_	2.34
		Cr_2_O_3_	<0.01
		V_2_O_5_	<0.01
		ZrO_2_	<0.02
		ZnO	0.04
		Loss on ignition	14.20

**Table 3:** Solubility test of wood ash in water.

	Wood Ash (g)	Mass Loss (g)	Material Dissolved (%)
1	14.10	0.90	6.30
2	15.00	1.20	8.00
3	14.30	0.90	6.30
		Average	6.90

**Table 4:** Consistency test results.

Mass Ratio	P0	P1	P2	P3	P4	P5
Wood ash/Cement	0.00	0.10	0.20	0.30	0.40	0.50
Wood/Binder	0.35	0.35	0.35	0.35	0.35	0.35
Water/Binder	1.00	1.04	1.08	1.12	1.16	1.20

**Table 5:** Average bending strength test results of wood-cement-ash particleboards (WCAP). Mean values with the same superscript are not significantly different for *p* = 0.05; standard deviation is given in parentheses.

		P0	P1	P2	P3	P4	P5
**3 days**	MOR (MPa)	0.92_(0.16)_	0.85_(0.04)_	0.75_(0.02)_	0.68_(0.07)_	0.53_(0.04)_	0.35_(0.08)_
	MOE (GPa)	1.04_(0.21)_	0.90_(0.21)_	0.84_(0.24)_	0.75_(0.08)_	0.58_(0.07)_	0.54_(0.08)_
**7 days**	MOR (MPa)	1.35_(0.21)_	1.28_(0.24)_	1.22_(0.17)_	1.15_(0.17)_	0.74_(0.05)_	0.43_(0.05)_
	MOE (GPa)	1.12_(0.14)_	1.12_(0.15)_	1.05_(0.13)_	1.01_(0.18)_	0.87_(0.03)_	0.70_(0.08)_
**28 days**	MOR (MPa)	1.36^(x)^_(0.32)_	1.30^(x)^_(0.33)_	1.24^(x)^_(0.21)_	1.20^(x)^_(0.16)_	0.78^(y)^_(0.25)_	0.47^z)^_(0.21)_
	MOE (GPa)	1.40^(a)^_(0.17)_	1.39^(a)^_(0.12)_	1.07^(b)^_(0.07)_	1.12^(b)^_(0.12)_	0.82^(c)^_(0.14)_	0.50^(d)^_(0.24)_

**Table 6:** Average water absorption and swelling of WCAP as a function of the WA content.

		P0	P1	P2	P3	P4	P5
Water absorption (%)	2 h	36.5	39.0	43.4	48.0	60.3	76.9
	24 h	38.8	42.7	47.5	52.0	61.6	76.1
Thickness swelling (%)	2 h	0.4	0.8	0.5	0.9	0.9	0.7
	24 h	2.0	0.9	0.7	1.6	1.6	0.8

**Table 7:** Average thermal properties and density of WCAP as a function of the WA content.

	P0	P1	P2	P3	P4	P5
**Specific gravity**	0.63	0.61	0.59	0.57	0.43	0.39
**Thermal conductivity (W/m·K)**	0.13	0.12	0.12	0.11	0.08	0.07
**Heat capacity (J/g·K)**	1304	1334	1368	1390	1424	1470
